# Optimizing the Outcome of Anti-Myeloma Treatment with Daratumumab

**DOI:** 10.3390/jcm10051002

**Published:** 2021-03-02

**Authors:** Torben Plesner

**Affiliations:** Department of Hematology, Vejle Hospital, Institute of Regional Health Science, University of Southern Denmark, Beriderbakken 4 DK, 7100 Vejle, Denmark; torben.plesner@rsyd.dk; Tel.: +45-7940-6313

**Keywords:** Daratumumab, multiple myeloma, immunoglobulin replacement therapy, line of therapy, duration of therapy, immunomodulation

## Abstract

A search of the scientific literature for *Daratumumab* and *myeloma* gives more than 600 results (January 2021), which reflects the interest and activity around this antibody, an interest that was also reflected by the assignment of breakthrough designation for Daratumumab as a treatment for multiple myeloma by FDA in 2013. The high expectations have been supported and met due to a very active clinical development program, and our insight into Daratumumab’s modes of action have been expanded by a concomitant, systematic activity of translational research. The scope of this article is to point to some areas where the outcome of treatment with Daratumumab for multiple myeloma may be improved with a focus on areas such as when to initiate treatment with Daratumumab, the use of supportive treatment, duration of therapy and some general thoughts about anti-myeloma treatment as a two-step process involving initial de-bulking followed by reprogramming of the host’s immune system and immune-mediated control of myeloma.

## 1. Introduction

This review is based on personal experience with Daratumumab for treatment of multiple myeloma for more than a decade, results from clinical studies and countless interactions with wonderful colleagues in the field. The intention is to point at some areas where the outcome of treatment may be further improved and to stimulate thoughts and discussions about areas of uncertainties with the hope of gaining optimal benefit from treatment with Daratumumab for our patients in the future.

## 2. Challenging a Dogma with Daratumumab

It is a well-established fact in myeloma that the number of patients that are offered a second or subsequent line of therapy is steadily declining and that the depth and duration of responses also decline with time. However, the introduction of Daratumumab for treatment of myeloma has challenged this dogma. In an update of the POLLUX trail, where Daratumumab was used in combination with lenalidomide and dexamethasone for treatment of relapsed/refractory myeloma, the outcome was far better than what could have been anticipated when comparing with the outcome of second line of therapy in the real world (Table 1) [1,2].

One may question the relevance of this comparison since in the real world many patients have comorbidities or elements of frailty that preclude their participation in clinical trials, but this problem is more evident in the case of first line treatment. Patients that make it to a second line of therapy in the real world have been selected for their ability to survive. In the present example of real world data, 20% of the patients did not make it to the second line of therapy [2].

## 3. Prolonging Survival without a Response

**Figure 1:**
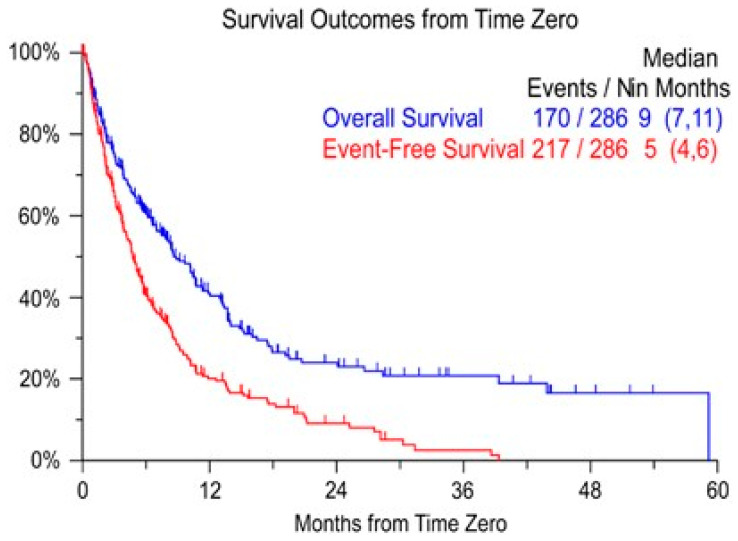
Survival after being refractory to bortezomib and relapsed/refractory or intolerant to an IMID [5].

The results of the first clinical trials (GEN501 and SIRIUS) testing Daratumumab as monotherapy of relapsed/refractory myeloma attracted a lot of interest [3,4]. A response rate of 31% was impressive for patients with advanced myeloma, and the fact that this could be obtained with an antibody was a break-through for myeloma therapy. The median overall survival of 20.1 months was more than a doubling of the 9-months survival that could be expected at that time for patients that were relapsing after treatment with bortezomib and an Immunomodulatory Drug (IMID) (Figure 1) [5].

**Figure 2:**
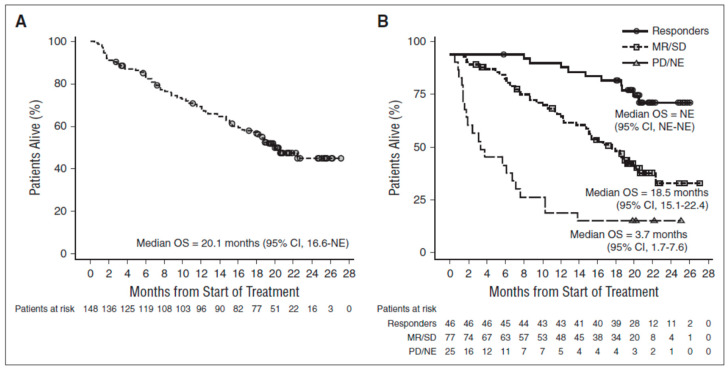
Overall survival of myeloma patients treated with Daratumumab monotherapy [6]. (**A**) All patients. (**B**) Patients stratified by response as Responders (Partial Response or better); Minor Response or Stable Disease (MR/SD) or Progressive Disease/Not Evaluable (PD/NE). Abbreviations: OS = Overall Survival; NE = Not Evaluable.

Perhaps of even greater interest was the additional finding that patients that did not obtain a response according to the International Myeloma Working Group (IMWG) criteria (52% of the study population) had a very substantial prolongation of their overall survival (median 18.5 months) (Figure 2) [6]. To understand this finding, one must take into consideration the many important modes of action that have been ascribed to Daratumumab. The immune mediated killing of myeloma cells by Complement Dependent Cytotoxicity (CDC), Antibody Dependent Cellular Cytotoxicity (ADCC) and Antibody Dependent Cellular Phagocytosis (ADCP) will presumably lead to a drop in the markers of active myeloma and thus a measurable response. On top of that, Daratumumab may contribute to a reprogramming of the host’s immune system and modulate the bone marrow microenvironment so that progression of myeloma is delayed and survival prolonged even in a setting without significant killing of myeloma cells (Table 2). Several modes of action by Daratumumab have been demonstrated including inhibition of production of immunosuppressive adenosine, inhibition of adhesion of myeloma cells to bone marrow stroma, inhibition of formation of nanotubes that transfer mitochondria from stroma cells to myeloma cells and invigorate the myeloma cells, direct stimulation of T-cell mediated cytotoxicity, inhibition of expression of immunosuppressive PD-L1 by antigen-presenting cells and elimination of regulatory cells of the T-, B- and monocyte/macrophage system that inhibit cytotoxic T-cells.

## 4. The Dubious Concept of High CD38 Expression Being Beneficial

Careful analysis of the outcome of the first clinical trials of single agent Daratumumab for treatment of relapsed/refractory myeloma revealed that patients with a high expression of CD38 by myeloma cells had a better chance of obtaining a response of PR or better [7]. This observation has fostered the assumption that high expression of CD38 is beneficial for the response to Daratumumab although the authors of the original publication pointed out, that obtaining long-term disease control with Daratumumab may differ in requirements from the rapid initial response that most likely is dependent on killing of myeloma cells by CDC, ADCC and ADCP. As a result, several strategies have been proposed to increase CD38 expression by myeloma cells in patients progressing on Daratumumab such as pharmacological intervention with ATRA or panobinostat or introduction of a treatment-free interval to allow for spontaneous recovery of CD38 expression with the hope of improving the response to treatment with Daratumumab [15,16]. However, these attempts may be contra-productive if CD38 is in fact a growth and survival factor for myeloma cells as suggested by the prolonged survival of patients obtaining only MR or SD during treatment with Daratumumab in a situation where CD38 expression by the myeloma cells is known to be reduced [7] (Figure 2), and when one take into consideration the modes of action of Daratumumab where either low expression of CD38 is beneficial or continued exposure to Daratumumab is important (Table 2). In fact, high expression of CD38 was not correlated to survival (PFS or OS), only to response in the trials of Daratumumab monotherapy [7].

## 5. Treat Early

Treatment with Daratumumab should be initiated as early as possible in the patient’s course of disease. A comparison of two recent trials, the MAIA study, where newly diagnosed, transplant non-eligible myeloma patients were treated, with the POLLUX trial that enrolled patients with relapsed/refractory myeloma, show a superior PFS at 30 months (71% versus 60%) although the patients enrolled in the MAIA study were considerably older (44% 75 years of age or older) compared with the POLLUX trial [17,18] (Table 3). The trial designs were similar with lenalidomide and dexamethasone as the backbone of both trials and Daratumumab was added in the experimental arms of the studies following the approved dosing and schedule for intravenous Daratumumab. Interestingly there was no difference between the trials regarding the chance of obtaining a high-quality response (sCR/CR, Minimal Residual Disease (MRD) negativity) so it seems to be the durability of response that is affected by delayed treatment. An important aspect of treating early with expensive drug combinations is the financial burden that is posed on the society [19].

## 6. Supportive Treatment

**Figure 3:**
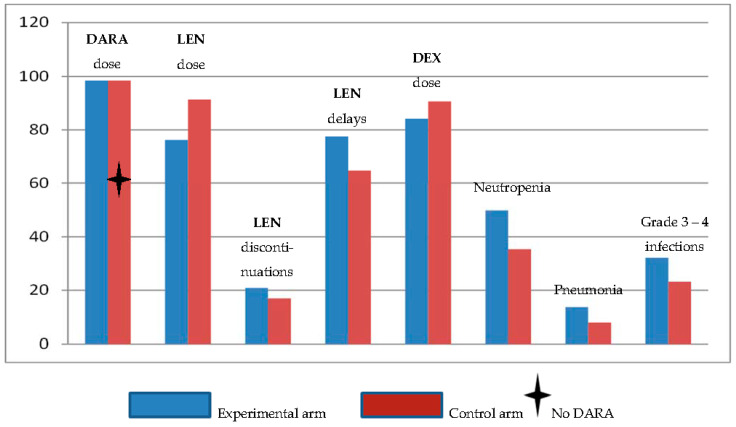
Dose intensity of Daratumumab, lenalidomide and dexamethasone, dose modifications of lenalidomide and adverse events in the MAIA trial [17].

Keep the patient on the track. Avoid dose-interruptions, dose reductions and complications that may cause delays of treatment, impaired quality of life and especially in the very elderly patient deterioration of performance status (i.e., infections). Ideally, Daratumumab is not a stand-alone drug except perhaps as maintenance after having obtained a very good response in combination with other anti-myeloma drugs. It is therefore a problem that lenalidomide was reduced in dose, discontinued or delayed in the experimental arm of the MAIA trial due to an increased risk of neutropenia and infections when Daratumumab was combined with lenalidomide and dexamethasone [17] (Figure 3). In most cases neutropenia can be prevented by use of G-CSF given one, two or three times per week as needed and thereby allow the use of full-dose lenalidomide without interruptions.

When it comes to prevention of infections, the situation is not so clear because we lack large-scale clinical trials that can guide us. However, a recent small Italian study of 46 patients showed a very significant reduction in the risk of infections by prophylactic treatment with immunoglobulin. The annual rate of infections in the untreated group was reduced from 24 episodes of septicemia, 18 episodes of bacterial pneumonias and 43 episodes of “bronchitis with septicemia” to none of these types of infections in the group that had received immunoglobulin prophylaxis [20]. Similar findings were reported in an almost ancient study of 83 myeloma patients randomized to receive immunoglobulin or placebo in a double-blind study [21]. Both studies have the draw back that they are small and that they did not provide immunoglobulin prophylaxis immediately after the diagnosis of myeloma where the patients are most vulnerable and probably would benefit most from prophylaxis against infections. In the absence of solid evidence from large-scale clinical trials, sound clinical judgement must be exercised and prophylactic treatment with immunoglobulin initiated early in patients at risk of serious complications.

## 7. Use the Best Treatment Upfront

**Figure 4:**
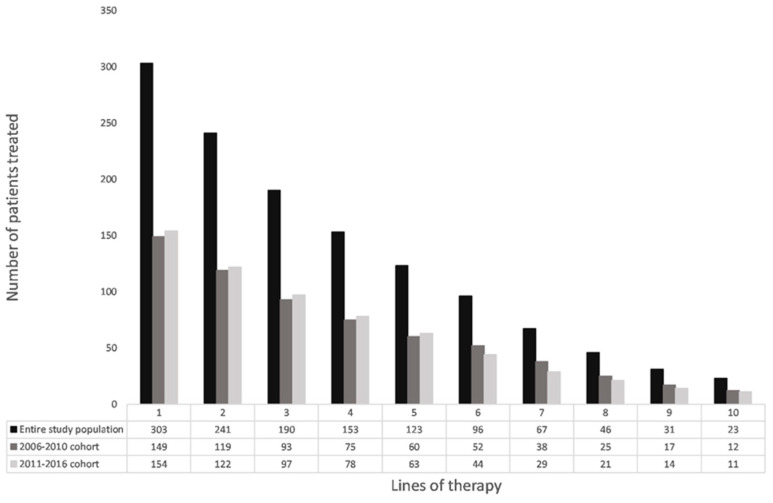
Number of myeloma patients receiving one or more lines of treatment in a single center (Vejle Hospital, Vejle, Denmark) [2].

Considering the exceptional outcome of the MAIA trial with 71% of the patients being alive without progression after 30 months durable responses may be obtained even in an elderly population of myeloma patients with very few side effects and a substantial improvement in the quality of life (Perrot et al. Journal of Clinical Oncology, in press 2021). Ongoing studies will clarify if addition of a proteasome inhibitor to the combination of Daratumumab and IMID will further improve the outcome. A substantial number of patients will not receive a second line of therapy and there is a steady decline in the number of patients offered second and subsequent lines of therapy (Figure 4), and in the duration of response after later lines of therapy [2].

Thus, within the limitations of country-specific guidelines and rules every effort should be made to offer a patient the best available treatment upfront. Although cross-trial comparisons are notoriously difficult to make it is interesting that the widely used, and for good reasons very popular VRd regimen, reported a PFS at 30 months of 64% with a median age of the study population of 63 years versus the MAIA trial with a PFS at 30 months of 71% and a study population with a median age of 73 [17,22].

## 8. Duration of Therapy

**Figure 5:**
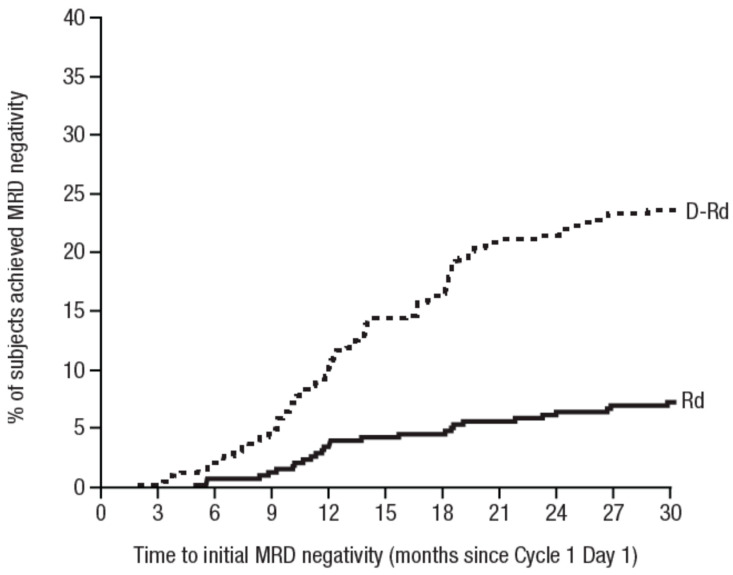
Time to initial MRD negativity in the MAIA trial [17]. Abbreviations: D-Rd = Daratumumab, Revlimide and Dexamethasone; Rd = Revlimide and Dexamethasone.

In the MAIA trial it was found that the rate of MRD negativity was increased by adding Daratumumab to a backbone of lenalidomide and dexamethasone. With a median follow up of 28 months the rate of MRD negativity continued to increase during 30 months of therapy suggesting that significant benefit may be obtained by continued treatment [17] (Figure 5). Prolonged treatment may be challenging due to the accumulation of side effects but since Daratumumab causes minimal if any long-term side effects it may be tempting to speculate if lenalidomide and/or dexamethasone may be omitted at some time point during therapy without compromising the efficacy of the treatment. An unpublished subgroup analysis of the POLLUX and MAIA trials suggests that this may indeed be the case. The numbers are small so the results should be interpreted with caution but the findings do support a strategy where lenalidomide and/or dexamethasone is stopped when the side effects pose a problem to the patient and impair the quality of life. A few patients from the early start of clinical trials with Daratumumab for relapsed/refractory myeloma (GEN501 and GEN503) are still receiving therapy for 6–7 years now for several years with single-agent Daratumumab without side effects or any evidence of recurrence of myeloma. However, since we have no stopping rules and do not dare to assume that the patients have been cured of myeloma, treatment is still ongoing.

## 9. Anti-Myeloma Therapy with Daratumumab: Re-Treatment or Continuous Therapy?

One may speculate why Daratumumab has been so successful in the treatment of myeloma. A plausible explanation may be that CD38 is a growth and survival factor for myeloma cells and that reducing CD38 expression and keeping it low at any time may inhibit proliferation and survival of myeloma cells. However, this is a matter of debate and the proponents of a *re-treatment* approach after having allowed for a recovery of CD38 expression by myeloma cells during a treatment free interval still have the momentum, as opposed to *continuous therapy* with Daratumumab over several lines of therapy. The clinical trials that may settle this debate are still lacking although a Daratumumab re-treatment trial has been initiated (NCT03871829), and also a trial that makes use of co-treatment with ATRA with the purpose of increasing the expression of CD38 by myeloma cells, thereby making them more sensitive to some of Daratumumab’s modes of action (CDC, ADCC, ADCP) (NCT02751255).

## 10. Frailty as an Issue

In recent years increasing focus has been directed towards the importance of frailty for the possibility to offer effective anti-myeloma therapy. Many patients are diagnosed with myeloma at a high age at a time point in their lives where aging and co-morbidities may pose significant challenges to anti-myeloma therapy. However, Daratumumab shows very little overlapping toxicity when used in combination with other anti-myeloma drugs. The most common finding is a slightly increased risk of neutropenia and infections as exemplified in Figure 3. In fact it was recently shown that addition of Daratumumab to Lenalidomide and Dexamethasone for first line treatment of an elderly population of myeloma patients in the MAIA trial resulted in a rapid and sustained improvement of the patient’s quality of life [23]. A retrospective analysis of patients enrolled into the MAIA trial according to frailty demonstrated that also the frail subgroup of patients achieved significant benefit from the addition of Daratumumab to Lenalidomide and Dexamethasone (manuscript submitted for publication January 2021).

## 11. Anti-Myeloma Therapy as a Two-Step Process

**Figure 6:**
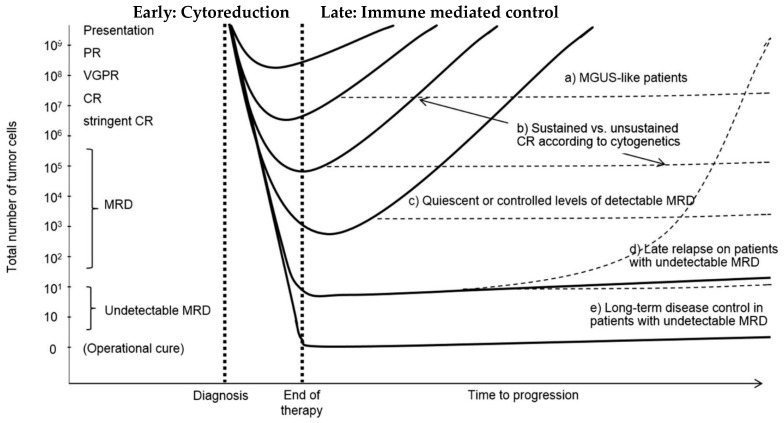
A hypothetical model proposing two phases of anti-myeloma therapy consisting of initial de-bulking followed by modulation of the bone marrow microenvironment and reprogramming of the host’s immune system resulting in a sustained disease control (modified from [24]). In situations where the initial de-bulking by chemotherapy is not followed by modulation of the bone marrow microenvironment and enhancement of the hosts’ immune system a rapid regrowth of tumor may occur. The figure was originally intended to illustrate the importance of obtaining and maintaining deep and sustained responses with MRD negativity.

If one accepts the notion that anti-myeloma therapy may be seen as a two-step process with (1) an initial killing of myeloma cells (de-bulking) followed by (2) alterations of the bone marrow microenvironment and reprogramming of the host’s immune system to allow for immune-mediated control of myeloma, and that both components are essential to obtain long-term disease control the reason for Daratumumab’s success becomes evident. Daratumumab can both kill myeloma cells by CDC, ADCC and ADCP, and reprogram the host’s immune system and change the bone marrow microenvironment in disfavor of the myeloma cells as reviewed in detail earlier. Thereby Daratumumab may contribute to both of the important parts of a successful anti-myeloma treatment (Figure 6).

The most important modes of action whereby Daratumumab contributes to the initial killing of myeloma cells may be by CDC, ADCC and ADCP. During the subsequent part of the treatment Daratumumab exerts its effects by eliminating regulatory cells of the T-, B- and Myeloid phenotype, direct stimulation of cytotoxic T-cells, inhibition of formation of immunosuppressive adenosine, inhibition of formation of nanotubes with concomitant transfer of mitochondria from stromal cells to myeloma cells, inhibition of adhesion of myeloma cells to stromal cells and inhibition of upregulation of PD-L1 induced by chemotherapy on antigen-presenting cells. The pleiotropic modes of action exerted by Daratumumab make it difficult to understand that patients may become truly refractory to all the aspects of Daratumumab’s effects. In most cases, Daratumumab cannot be seen as a stand-alone drug and the effect of Daratumumab is highly dependent on the simultaneous use of a suitable partner or partners. Clinical trials that can inform us if continued treatment with Daratumumab adds any benefit over several lines of therapy with a successive exchange of partner drugs are greatly needed.

## Figures and Tables

**Table 1:** Comparison of patient populations and response to second line therapy of myeloma.

Clinical Setting	Real World [2]	Pollux Trial [1]
Number of patients	303	286
Median age (years)	69	65
Age range (years)	30–90	34–89
Prior lines of therapy	1	1 (median)
Overall Response Rate %	59	93
Complete Response/Stringent Complete Response	17	57
Progression Free Survival (months)	10	45
Time to Next Treatment (months)	6	51

**Table 2:** Immune mediated killing of myeloma cells, reprogramming of the host’s immune system and modulation of the bone marrow microenvironment by Daratumumab.

Impact of CD38 Expression by Myeloma Cells	Mode of Action	Reference
High expression beneficial		
	CDC	[7]
	ADCC	[7]
	ADCP	[8]
*Low expression beneficial*		
	Inhibition of adenosine production	[9]
	Inhibition of adhesion to stroma	[10]
	Inhibition of nanotube formation	[11]
*Indeterminate but continuous exposure to Daratumumab beneficial*		
	Enhancement of T-cell mediated cytotoxicity	[12]
	Reduced PD-L1 expression by antigen-presenting cells	[13]
	Elimination of regulatory T-cells, B-cells and Myeloid cells	[14]

**Table 3:** Results of treatment with Daratumumab, lenalidomide and dexamethasone.

Trial Name	MAIA	POLLUX
Target population	NDMM	RRMM
Number of patients	368	286
Follow-up (months)	28	25
Median age (years)	73	65
Median number of prior lines of therapy	0	1
PFS at 30 months	71%	60%
sCR/CR rate	48%	51%
MRD negative rate (10^−5^)	24%	26%

Abbreviations: NDMM = Newly Diagnosed Multiple Myeloma; RRMM = Relapsed Refractory Multiple Myeloma; PFS = Progression Free Survival; CR/sCR = Complete Response/Stringent Complete Response; MRD = Minimal Residual Disease.

## Data Availability

Further information may be retrieved from the author.

## Abbreviations

CDC	Complement Mediated Cytotoxicity
ADCC	Antibody Dependent Cellular Cytotoxicity
ADCP	Antibody Dependent Cellular Phagocytosis
PFS	Progression Free Survival
OS	Overall Survival
CR	Complete Response
sCR	Stringent Complete Response
PR	Partial Response
